# A model invalidation-based approach for elucidating biological signalling pathways, applied to the chemotaxis pathway in *R. sphaeroides*

**DOI:** 10.1186/1752-0509-3-105

**Published:** 2009-10-31

**Authors:** Mark AJ Roberts, Elias August, Abdullah Hamadeh, Philip K Maini, Patrick E McSharry, Judith P Armitage, Antonis Papachristodoulou

**Affiliations:** 1 Control Group, Department of Engineering Science, University of Oxford, Parks Road, Oxford, OX1 3PJ, UK; 2 Department of Biochemistry, University of Oxford, South Parks Road, Oxford, OX1 3QU, UK; 3 Oxford Centre for Integrative Systems Biology, Department of Biochemistry, University of Oxford, South Parks Road, Oxford, OX1 3QU, UK; 4 Centre for Mathematical Biology, Mathematical Institute, University of Oxford, 24-29 St Giles', Oxford, OX1 3LB, UK; 5 Mathematical Institute, University of Oxford, 24-29 St Giles', Oxford, OX1 3LB, UK

## Abstract

**Background:**

Developing methods for understanding the connectivity of signalling pathways is a major challenge in biological research. For this purpose, mathematical models are routinely developed based on experimental observations, which also allow the prediction of the system behaviour under different experimental conditions. Often, however, the same experimental data can be represented by several competing network models.

**Results:**

In this paper, we developed a novel mathematical model/experiment design cycle to help determine the probable network connectivity by iteratively invalidating models corresponding to competing signalling pathways. To do this, we systematically design experiments *in silico *that discriminate best between models of the competing signalling pathways. The method determines the inputs and parameter perturbations that will differentiate best between model outputs, corresponding to what can be measured/observed experimentally. We applied our method to the unknown connectivities in the chemotaxis pathway of the bacterium *Rhodobacter sphaeroides*. We first developed several models of *R. sphaeroides *chemotaxis corresponding to different signalling networks, all of which are biologically plausible. Parameters in these models were fitted so that they all represented wild type data equally well. The models were then compared to current mutant data and some were invalidated. To discriminate between the remaining models we used ideas from control systems theory to determine efficiently *in silico *an input profile that would result in the biggest difference in model outputs. However, when we applied this input to the models, we found it to be insufficient for discrimination *in silico*. Thus, to achieve better discrimination, we determined the best change in initial conditions (total protein concentrations) as well as the best change in the input profile. The designed experiments were then performed on live cells and the resulting data used to invalidate all but one of the remaining candidate models.

**Conclusion:**

We successfully applied our method to chemotaxis in *R. sphaeroides *and the results from the experiments designed using this methodology allowed us to invalidate all but one of the proposed network models. The methodology we present is general and can be applied to a range of other biological networks.

## Background

Understanding the connectivity of signalling pathways within organisms has always been an important challenge in biological research. One approach to address this is to study individual parts *in vitro *and look at protein localisation, homologies and co-expression in order to elucidate signalling pathway connectivity. Another approach is to use genetics and mutants to attempt to work out the pathway connectivity *in vivo*.

More recently, systems biology approaches have used quantitative measurements to develop mathematical models that can be used for understanding the properties of biological signalling pathways and their connectivity [1-3]. These models are usually a result of cyclic mathematical model/experiment design iterations, which aim to yield maximum information about the system under study [4,5]. It is well known, however, that models of comparable complexity corresponding to different pathway connectivities may fit experimental data equally well, leaving the researcher with the dilemma of which model is correct [6]. The question of which model is correct is actually impossible to answer as model validation is a misnomer. The related question of which models are invalid can be answered if appropriate data are available, i.e. the inability of a model to reproduce a data set renders it invalid. Applied in this way, model invalidation can be used to reduce the number of possible models [7], hence narrowing down the number of possible network connectivities.

Previous work in model invalidation emphasised the importance of using time-varying inputs to the system under study. One method of invalidation is to apply a dynamical input and try to maximise the difference in the phase shift of two competing deterministic models [8]. The disadvantage of this approach is that phase-shift is usually difficult to quantify, especially with noisy data. Another approach is presented in [9], where the authors develop a dynamic model-based controller and an input profile that drives the system output along a prescribed target trajectory. However, this approach requires the implementation of a controller in the laboratory which may prove difficult. Other approaches for model invalidation are presented in [5,10,11], and the references therein. These lack-of-fit methods are used to invalidate models in a statistical manner, but there can be problems with this approach as it relies on large data sets and focuses on obtaining reliable parameter estimates rather than network connectivities. There is also the issue that a wide range of model parameters could give a very similar model output and these methods would have difficulty coping with this.

In this paper we present a new method for developing mathematical models of biological signalling networks, aiming to understand biological network structure. Given a set of experimental data, models corresponding to competing network connectivies are first constructed, all of which can explain wild type data equally well. Then, experiments which maximally discriminate between models corresponding to different networks are designed systematically. These experimental results are used to invalidate models of these networks, resulting in a cyclic process which aims to produce a mathematical model corresponding to a signalling pathway structure which explains all available wild type and mutant experimental data. Our method is applicable to biological pathways for which it is possible to experimentally modify the input profile and measure the output simultaneously. One such system is the chemotaxis signalling pathway, for which tethered cell experiments can be performed in a flow cell and used to measure the response of the system to dynamic ligand concentration profiles.

Chemotaxis is the biasing of movement towards regions of higher concentrations of beneficial or lower concentrations of toxic chemicals by altering the frequency of flagella switching [12]. The signalling pathway within *E. coli *is well understood and is a simple circuit with one feedback loop [13]. The receptor in the system is a Methyl-accepting Chemotaxis Protein (MCP) that senses ligands outside the cell. Associated with the MCP is the histidine protein kinase called CheA. Binding of certain ligands to MCP decreases the auto-phosphorylation rate of CheA. CheA can transfer phosphoryl groups to two possible response regulators, CheY and CheB. CheY-P interacts with motor binding sites of the multiple *E. coli *flagella motors causing a change in direction. The receptors are constantly methylated by the action of a methyltransferase CheR, while CheB-P acts as a methylesterase to demethylate the receptor, making it less responsive to ligand binding. This creates a feedback loop, allowing for adaptation. Adaptation allows *E. coli *to react to changes of the concentration gradient of ligands and not to changes in concentrations *per se*. Finally, CheZ acts to dephosphorylate CheY and to terminate the signal.

**Figure 1:**
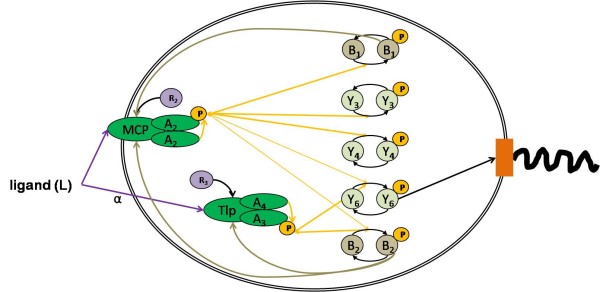
**The chemotaxis pathway of *R. sphaeroides***. The currently known localisation and connectivity of the chemotaxis pathway in *R. sphaeroides *is shown. The localisation of the two signalling clusters, one at the pole and one in the cytoplasm was determined by GFP fusions and immunoflouresence [15]. The remaining connectivities, was determined be *in vitro *phosphotransfer measurements and by mutant data [16,21]. It is currently unknown what role CheY_3 _and CheY_4 _perform in the system.

Chemotaxis pathways in other species are less well characterised and often contain multiple homologues of the *E. coli *system and sometimes proteins not found in the *E. coli *signalling pathway, for example, CheD [13]. Chemotaxis pathways in other species may also have a different connectivity - for example in *S. meliloti*, only one of the two CheY homologues interacts with the motor, with the other CheY acting as a phosphate sink [13,14]. Another good example is the chemotaxis pathway in *R. sphaeroides*. This bacterium has three chemotaxis operons, two of which are expressed under normal laboratory conditions. Proteins expressed from these operons have previously been shown to localise to discrete signalling clusters, one at the poles of the cell, similar to *E. coli*, and one in the cytoplasm [15]. This localisation is thought to prevent cross talk between the two clusters, allowing them to potentially signal separately. Despite these data and corresponding data on the possible phosphotransfer patterns [16], the way the signal is transmitted and integrated between the chemotaxis clusters to control flagella activity is currently unknown [17]. This is a good example of the difficulty of inferring a network structure from homology alone. The current known connectivity and protein localisation is shown in Figure 1.

In this paper, we apply our method of model invalidation to different possible and plausible connectivity structures in the chemotaxis pathway of *R. sphaeroides*. In particular, we use the model invalidation/experiment design cycle outlined above, in order to shed light on the signalling pathway in *R. sphaeroides*.

## Results

### A method for discriminating between competing network models

A general method for designing experiments so as to render the outputs of candidate models as different as possible is described. These experiments can then be performed in the laboratory and, when compared to the model predictions, allow the invalidation of some of the candidate models, even when the experimental data set is noisy.

Our method involves the development of ODE models corresponding to different signalling pathway connectivities, all of which can explain current wild type experimental data equally well. The models have in common all currently known interactions and differ in that each model represents a new speculative pathway connectivity. Some parameters in these models are known and some others are unknown. Thus in principle, one can develop a "set" of models with uncertain parameters for a particular signalling structure, each member of this set representing the wild type experimental data equally well. We would want to discriminate between these "sets" of models (which represent signalling pathway structures) but since this is a very hard problem, our method uses a nominal model from each set and then designs an experiment in order to discriminate between these nominal models. Once the discriminatory experiments between the nominal models are designed *in silico*, we *a posteriori *assess the discriminatory property of the stimulus by simulating the behaviour for many, randomly chosen, models within these sets in order to see whether the outputs from the two model sets remain distinguishable.

The discriminatory experiments were designed by initially determining the input profile that maximises the magnitude of the squared output difference between models over time. This is typically an optimal control problem, which is often laborious to solve and can result in an input profile that is difficult to realize experimentally [18]. In order to design the input profile in a numerically efficient manner, we used the following result from linear systems theory: for a linear time-invariant system, the input that produces the largest energy output given an input of fixed energy is a (truncated) sinusoid of a particular frequency [19]. The frequency can be obtained using a Bode plot (see Methods section), a tool which is often used in control and systems theory [20]. Thus, we first linearised the models and then determined this particular frequency of a sinusoidal input corresponding to the error system, i.e. the difference between the two models.

If the above method is insufficient to discriminate between the models then a further method of 'mutating' the two models in biological terms or changing parameters/initial conditions (e.g. alter the initial protein concentrations) in engineering terms is applied to achieve discrimination. These changes can be tested *in silico *to determine those which will discriminate best between the models under test, before undertaking experiments. The exact nature of the perturbation that can be performed will vary with the system being investigated, but could include altering protein levels by knockout, knockdown (e.g. RNAi in eukaryotes), protein over expression, etc. Thus the space of perturbation which can be searched will be defined by what is possible to implement experimentally in the biological system under investigation.

The designed experiments can then be implemented and the data obtained from such laboratory experiments used to help us differentiate between the models under study, by invalidation. In the above procedure we used deterministic models and a worst-case input design procedure, therefore even if the data are noisy, we expected that we would still be able to invalidate some of these models.

### Determining pathway connectivity in *R. sphaeroides *chemotaxis

We applied our model invalidation method to elucidate unknown interconnections within the *R. sphaeroides *chemotactic signalling pathway. *In vitro *phosphorylation measurements have determined some of the internal connectivity and genetic work has shown that only CheY_6 _and either CheY_3 _or CheY_4 _are required for motor switching and that CheY_6 _can bind to the motor [21] (Figure 1). Whilst it has been shown *in vitro *that all CheYs _1-6_ can interact with FliM, the rotor switch [22], it is currently unknown whether CheY_3 _and CheY_4 _proteins cause flagella motor switching or whether some CheYs have an effect on other parts of the signalling pathway to influence the motor indirectly, for example, by acting as a phosphate sink.

### Model creation and parameter estimation

**Figure 2:**
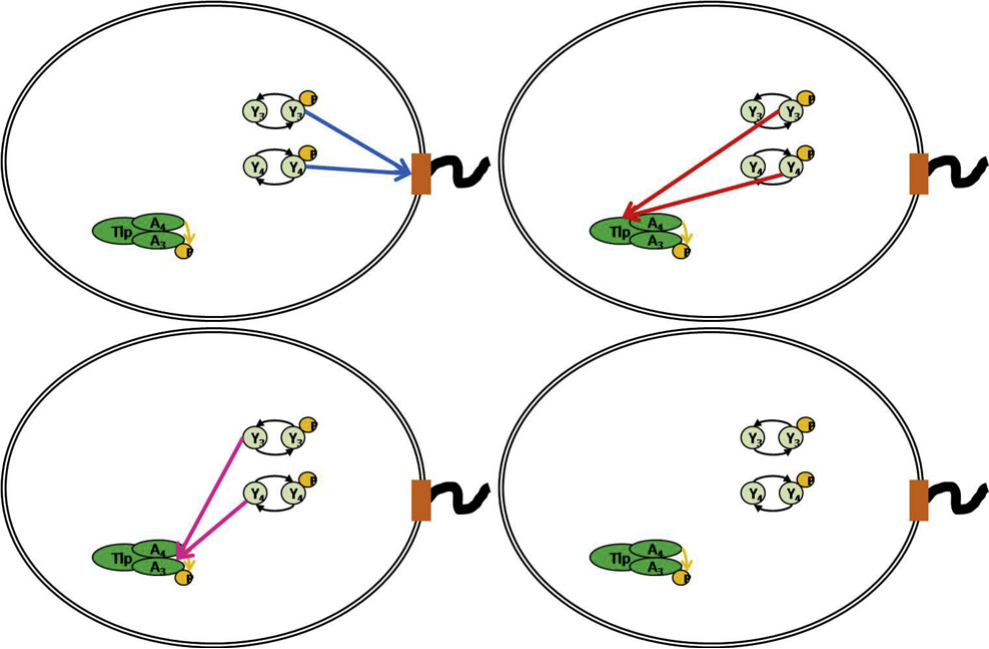
**The models of the *R. sphaeroides *chemotaxis system**. The different connecitvities modelled for CheY_3 _and CheY_4 _are shown in this diagram. The coloured lines represent three models presented in this paper. In the blue model CheY_3_-P and CheY_4_-P bind to FliM in the motor co-operatively with CheY_6_-P. In the red model CheY_3 _and CheY_4 _form a connection with the cytoplasmic cluster and act antagonistically to ligand in altering the kinase activity of CheA_4_; therefore, the increase in CheY_3/4_-P acts to increase the rate of CheA_3 _phosphorlyation by CheA_4_. In the magenta model CheY_3/4 _interact with CheA_3 _preventing CheY_6 _binding and hence phosphotransfer. The fourth model corresponds to a model, in which CheY_3 _and CheY_4 _act as phosphate sinks only and hence do not interact with anything. This is referred to as the grey model in this paper. It should be noted that the other three models also contain this interconnection and that the difference in the grey model is that only this connection exists.

**Figure 3:**
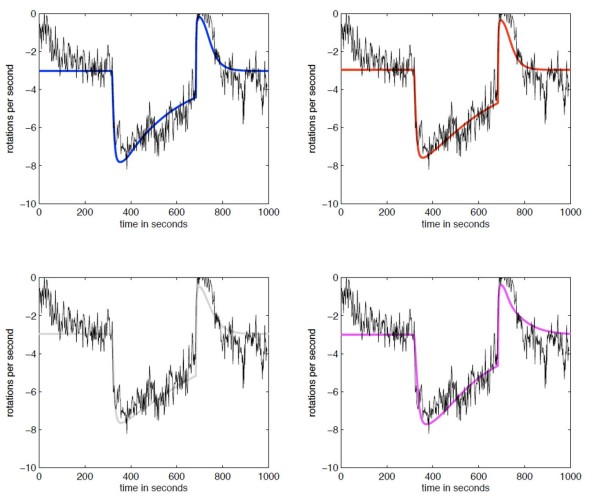
**Fitting model parameters**. The three parameters *K*_1 _- *K*_3 _are unknown and thus the models were fitted to wild type data in order to obtain them. WS8N (wild type) was tethered and recorded for 5 mins without, 5 mins with and 5 mins without 100 *μ*M propionate. The coloured lines represent the best fit of each model to this data by varying the parameters K_1-3 _using the methods described in Methods section.

We created a number of different models representing various plausible CheY_3_, CheY_4 _connectivities within the chemotaxis system (Figure 2; Methods; SMBL in Additional File 1). Each of these models contains all the currently known connectivities, shown in Figure 1, but differs in which unknown connectivity is considered. To allow each of our models to represent current, wild type observations equally well, we fitted the unknown parameters of each model (K_1-3_) such that the error between model prediction and wild type data is small (Figure 3). These parameters represented the activation of the receptor on the CheA (K_1_), and the effect of and rate of methylation on the receptor (K_2,3_). These values are currently unknown biologically. The fitting results in the models with different connectivities having different such parameter values.

We obtained the following values for the parameters for the different models (the units for values of *K*_1 _are in *μ*M, and in  for *K*_2 _and *K*_3_):

• blue: *K*_1 _= 1, *K*_2 _= 16.5 and *K*_3 _= 0.015

• red: *K*_1 _= 25, *K*_2 _= 3250 and *K*_3 _= 0.075

• grey: *K*_1 _= 1, *K*_2 _= 97.5 and *K*_3 _= 0.0023

• magenta: *K*_1 _= 1, *K*_2 _= 0.4 and *K*_3 _= 0.011

We assumed that these parameters are the same for the polar and cytoplasic clusters, so .

We used the Pearson product-moment correlation coefficient as a measure of correlation between model prediction and data. For 200 ≤ *t *≤ 800 seconds, we found a good correlation between data and the predictions made by the models. For example, we obtained a coefficient of 0.9466 for the blue model and of 0.9544 for the red model.

**Figure 4:**
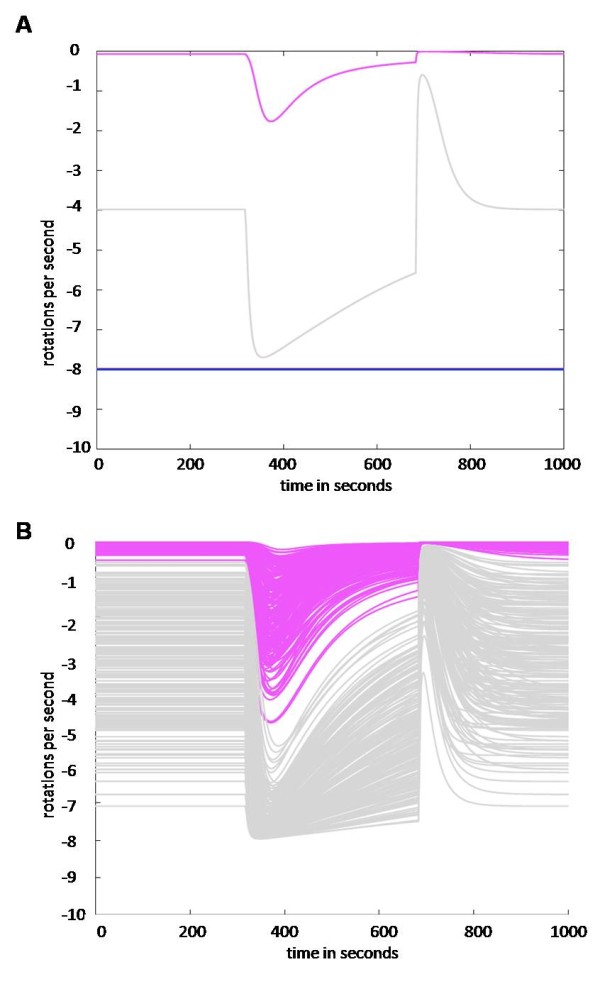
**Simulation of ΔCheY_3 _Y_4_**. A] Models representing the mutants lacking CheY_3 _and CheY_4 _were simulated. The grey and the magenta model are chemotactic (they still respond to stimulus) under these conditions and the steady state of magenta model is always close to zero. These results do not correspond to experimental observation. B] The robustness of our invalidation of the grey and magenta models was tested by running 200 simulations of the model lacking CheY_3 _and CheY_4_, allowing parameters *k*_1 _- *k*_14 _to vary by ± 50%. None of these changes produced a non-chemotactic model.

To ensure our models can fit all current experimental data we then compared the output of all models to data previously determined for the deletion of various genes within *R. sphaeroides *[21]. One possible plausible connectivity contains CheY_3 _and CheY_4 _acting only as phosphate sinks, similar to the roles of the multiple CheY's in *S. meliloti *[13,14]. However, the model representing this connectivity (grey model) was unable to fit mutant experimental data as it still showed chemotaxis in a CheY_3_Y_4 _deletion state (ΔCheY_3_Y_4_). Another possible connectivity where CheY_3 _and CheY_4 _can bind to CheA_3 _preventing CheY_6 _binding and hence phosphorylation was also considered (magenta model). However, this connectivity was unable to fit experimental data as it remained chemotactic in a ΔCheY_3_Y_4 _state (Figure 4A). To strengthen our conclusion that both connectivities are invalid, we ran 200 simulations of the models lacking CheY_3 _and CheY_4_, in which we allowed parameters *k*_1 _to *k*_14 _(Table 1) to vary by ± 50%. We observed that, even when we allowed for greatly different parameter configurations, the connecitvities modelled by the magenta and grey models are still chemotactic, hence our invalidation is robust (Figure 4B). As opposed to the grey and magenta model, the red and the blue model cannot be invalidated by above deletion data.

**Table 1:** Reaction rates used in the *R. sphaeroides *chemotaxis models

reaction	parameter(s)	value(s)
(R_1_) *A*_2 _→ *A*_2*p*_	*k*_1_	0.03 s^-1^

(R_2_) *A*_2*p*+ _*B*_1 _⇌ *A*_2 _+ *B*_1*p*_		0.035(*μ*Ms)^-1^, 0.01(*μ*Ms)^-1^

(R_3_) *A*_2*p *_+ *Y*_3 _⇌ *A*_2 _+ *Y*_3*p*_		0.065 (*μ*Ms)^-1^, 0

(R_4_) *A*_2*p *_+ *Y*_4 _⇌ *A*_2 _+ *Y*_4*p*_		0.004(*μ*Ms)^-1^, 0

(R_5_) *A*_2*p *_+ *Y*_6 _⇌ *A*_2 _+ *Y*_6*p*_		0.0006(*μ*Ms)^-1^, 0

(R_6_) *A*_2*p *_+ *B*_2 _⇌ *A*_2 _+ *B*_2*p*_		0.0035(*μ*Ms)^-1^, 0.01(*μ*Ms)^-1^

(R_7_) *B*_1*p *_→ *B*_1_	*k*_7_	0

(R_8_) *Y*_3*p *_→ *Y*_3_	*K*_8_	0.08s^-1^

(R_9_) *Y*_4*p *_→ *Y*_4_	*K*_9_	0.02s^-1^

(R_10_) *Y*_6*p *_→ *Y*_6_	*K*_10_	0.1s^-1^

(R_11_) *B*_2*p *_→ *B*_2_	*K*_11_	0.015s^-1^

(R_12_) *A*_3_*A*_4*p *_+ *Y*_6 _⇌ *A*_3_*A*_4+ _*Y*_6*p*_		0.1 (*μ*Ms)^-1^, 0

(R_13_) *A*_3_*A*_4*p *_+ *B*_2 _⇌ *A*_3_*A*_4 _+ *B*_2*p*_		0.006(*μ*Ms)^-1^, 0.07(*μ*Ms)^-1^

(R_14_)*A*_3_*A*_4 _→ *A*_3_*A*_4*p*_	*k*_14_	0.02s^-1^

The reaction rates used for the models determined by fitting phosphotransfer *in vitro *experiments [16,31]. For some of these reaction rates the best fit is zero suggesting that these reactions do not occur *in vitro*.

**Figure 5:**
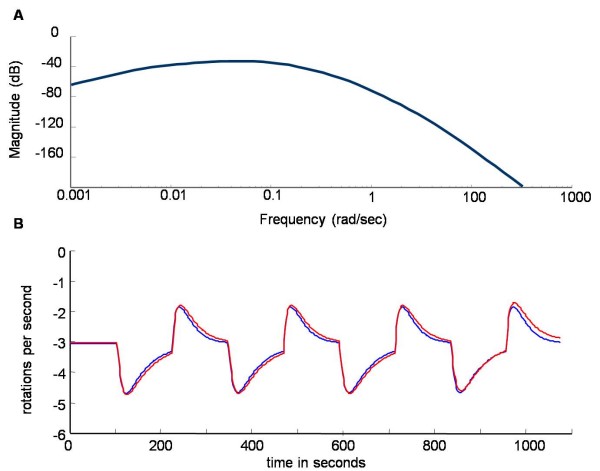
**Model discrimination by optimal frequency**. A] Bode plot determining the optimal input frequency for model discrimination. The Bode magnitude plot shows the output amplitude gain, *g*_*ω*_, versus frequency *ω *(in decibel; that is in 20 log(*g*_*ω*_)). Here, the gain corresponds to the ratio of the difference between the outputs of the blue and the red model, and the input amplitude (sinusoidal input); in other words, the maximum denotes at which frequency one can expect to see the largest difference between the models. The aim was to determine the optimal input frequency to apply to the system. This was determined to have a period of 4 minutes. B] Outputs of the simulation for the behaviour of the models with this optimal frequency applied as the input frequency demonstrating that the best input perturbation alone is not enough to discriminate between the two models. The coloured lines represent the models defined in Figure 2.

We then designed an experiment to invalidate one or both of the remaining two models following the model-invalidation cyclic procedure described in the background section. As mentioned previously, we chose a sinusoidal input with a particular frequency to help discriminate between the models. Using a Bode plot showing the response of the difference of the outputs to a sinusoidal input with fixed amplitude, we determined an input frequency (in terms of frequency of 100 *μ*M amplitude ligand application) in order to discriminate best between the remaining two models (Figure 5A). We chose an input period of four minutes, which was both close to the optimal in terms of discrimination and easy to implement experimentally. We then ran simulations of the time evolution of the models with alternating step inputs mimicking a sinusoidal input of this period (as this mirrors what can be implemented experimentally) to ensure that the difference in the outputs was sufficiently large to be accurately measured experimentally (Figure 5B).

We observed that the difference between the outputs of the two models under consideration was insufficient to allow discrimination between the models experimentally using this discrimination technique. Therefore, we sought experimental perturbations which resulted in a larger difference between the outputs of the different models. Possible experimental perturbations in this system involve deletions of one or more components, over expression or under expression of a protein component and growth of the bacteria in different growth conditions; the latter results in large-scale changes of the expression of the chemotaxis operons and hence protein concentrations.

**Figure 6:**
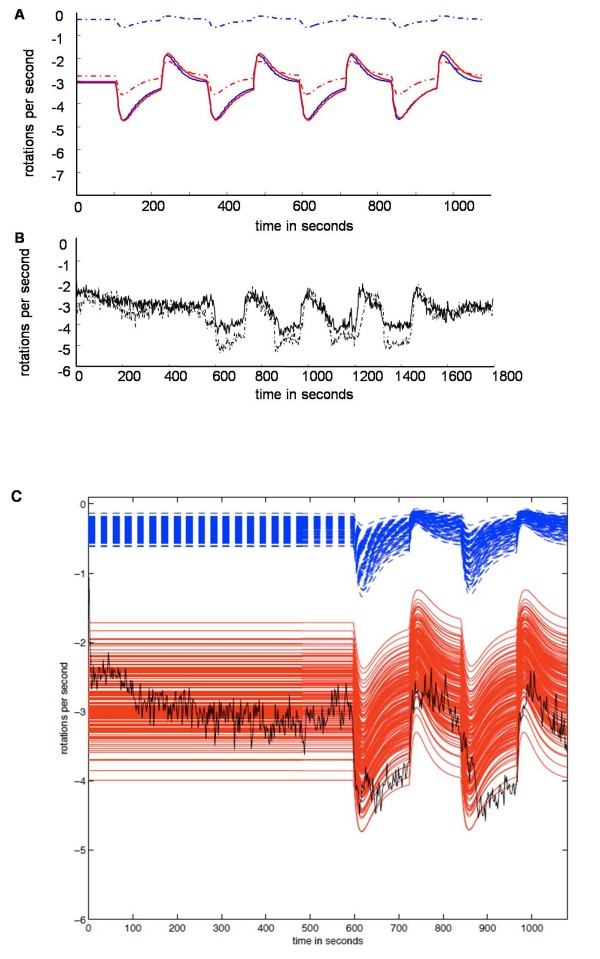
**Discrimination by optimal initial conditions**. A] Outputs of the simulations of the models with wild type (continuous lines) or levels of CheY_4 _(total) increased to 5-times normal levels at the start of the simulation (dotted lines). The coloured lines represent the models defined in Figure 1. B] Experimental outputs from tethered cell experiments, wild type output (continuous line) and CheY_4 _over-expressed in the cells at an average of 5-times the concentration found in wild type cells (dotted line). C] Robust invalidation of the blue model. To ensure that our invalidation was robust we ran 500 simulations assuming that CheY_4 _is 5-fold over-expressed. We vary parameters *k*_1 _**- ***k*_14 _by ± 15% and still observe the same clear difference between the behaviour of the blue and of the red model.

Before experimental implementation, we tested the possible perturbations *in silico*. The over expression of CheY_4 _was chosen as this resulted in a large difference in both the models' steady states and their dynamical behaviour (Figure 6A). Moreover, in order to ensure that this discrimination is not influenced by errors in determining parameter values, we confirmed that our findings are relatively robust to parameter changes. We allowed all parameters to vary by ± 15% and still observed the same clear difference in the behaviour of the models (Figure 6C).

We then used IPTG to experimentally induce the over expression of CheY_4 _from an expression plasmid. The level of CheY_4 _over expression as a population average was verified using semi-quantitative western blotting (data not shown). We observed that when CheY_4 _is over-expressed to five times the wild type level the tethered cell trace was similar to that of wild type cells (Figure 6B); this suggested that the model, in which the CheY_6 _and CheY_3 _or CheY_4 _bind to the motor co-operatively (blue model) and do not have other interactions, is invalid, as it is unable to represent our new experimental data (Figure 6B). Since the red model, where there is an interconnection between CheY_3 _and CheY_4 _and the cytoplasmic cluster altering the CheA_4 _kinase activity, can represent this data, it is the only model we cannot invalidate (Figure 6B).

## Discussion

We have developed a methodology for elucidating biological signalling pathways which we applied to understanding the chemotactic signalling pathway in *R. sphaeroides*. Our method differs from many other methods currently in use in that is based on manipulation and observation of the entire biological system under *in vivo *conditions and, importantly, offers a systematic approach to model invalidation based on cyclic model development/experiment design iterations. In particular, it does not aim at designing experiments for the refinement of parameter values, but rather at identifying possible interconnection structures. By doing so, our method considers a model representing a specific structure that it aims to invalidate.

We applied our method to a real biological system and successfully managed to invalidate some potential signalling pathway network models. Thus, this method helped to more rationally consider the interconnectivity of the chemotactic signalling pathway in *R. sphaeroides *through relatively simple input-output experiments *in vivo *and helps to design rational future experiments.

The method is applicable to other biological systems but requires an experimental setup where it is possible to control the input and measure the output simultaneously. Even with this limitation the method could be used for both pathway determination and parameter determination, where multiple models with different parameters could be systematically invalidated. Our method could potentially be used to help annotate and understand signalling pathways in non-model organisms, using the information from model organisms as a guide for the first model generation step. These could then be tested and models invalidated. A parameter search could also be performed by creating models which deviate from the model organism data and then, through model invalidation, determining which parameter sets are able to fit the experimental data. In all these cases though, the input of the system must be under experimental control and the output easily measured.

A potential limitation would be the case in which two models from different network structures produce outputs that are indistinguishable under all potential experimental conditions. The problem in this case could lie with the particular properties of the network and their discriminatory nature - i.e., whether such differences in structure are identifiable from input-output experiments [23]. Performing all calculations *in silico *before any experiments are undertaken is important in saving experimental time ensuring that experiments are only performed when the results will have discriminating property between the models under test.

In the system studied in this paper the modelling and experiment design was done on average cell traces for *R. sphaeroides *responses. The reason for this is that single cell behaviour is noisy, and in any case the model parameters we have available at the start of the procedure correspond to the average system behaviour. The total protein concentrations, for example, are the population average determined for these growth conditions. Thus the model output is for the 'average' cell hence we compared the output to the average of the tethered cell data.

When constructing the models we used *in vitro *rates as it is very difficult, if not impossible to measure these rates *in vivo*. The multiple homologues in *R. sphaeroides *would prevent a FRET assay such as the one applied to *E. coli *being used. Thus the parameters are the best estimate of the real value. To allow for this uncertainty we considered models where these parameters were varied to ensure robust invalidation. Also for simplicity in the modelling we did not consider the increased concentration of the CheAs at specific points in the cell due to clustering nor the effects of clustering. This is because the exact concentration of CheA in specific regions and the effects of clustering are currently impossible to measure experimentally. The fitted parameter K_1_, which relates the activation of the ligand on the receptor, includes the effect of clustering and as such it is accounted for in our models. To ensure that this is robust we also allowed the concentration of the CheA proteins to vary sufficiently and our invalidation conclusion was still correct.

Interestingly, the connectivity that best fits the experimental data suggests that in *R. sphaeroides *CheY_3_-P and/or CheY_4_-P do not bind co-operatively with CheY_6 _to FliM. The model we have been unable to invalidate suggests that CheY_3_-P and CheY_4_-P form a link between the polar and cytoplasmic signalling clusters, helping transmit the signal between the two clusters. We have through modelling and comparing to experimental data invalidated a model representing the previously held hypothesis that CheY_3 _and CheY_4 _act only as phosphate sinks for the system. We have also invalidated a model with strict co-operative binding of CheY's at the motor and as such our technique adds to the body of knowledge on *R. sphaeroides *chemotaxis.

## Conclusion

In this paper we have developed a control engineering method for elucidating biological signalling pathways and applied it to a real system. This method is based on multiple model creation and subsequent invalidation using *in silico *designed experiments. This is a general method that can be applied to other biological pathways where it is possible to control the input and measure the output in simultaneously. We used the method to invalidate all but one model for the chemotaxis signalling pathway in *R. sphaeroides *and in doing so have invalidated models of certain possible connectivities.

## Methods

### Strains and growth conditions

Bacterial strains and plasmids are listed in Table 2. *R. sphaeroides *strains were grown aerobically in succinate medium [24] at 30°C with shaking. When appropriate, the antibiotics nalidixic acid and kanamycin were used at 25 *μ*gml^-1^.

**Table 2:** Strains and plasmids

Strain/Plasmid	Characteristics	Source
*R. sphaeroides *WS8N	Spontaneous nalidixic acid resistant mutant of wild type WS8	[34]

*R. sphaeroides *JPA421	WS8N with the *cheY*_4 _gene deleted by genomic replacement	[35]

*E. coli *S17-1 *λ*pir	A strain capable of mobilising pAE and pAY4 into *R. sphaeroides*, Sm^R^	[36]

Plasmids		

pIND4	Over expression vector with a *lac *inducible promoter, capable of replication in *R. sphaeroides*	[25]

pIND4-Y4	pAE containing *cheY*_4_	[29]

Strains and Plasmids used in this study

### Protein over expression

pIND4-Y4 was transformed into S-17 and conjugated into *R. sphaeroides *as described previously [25]. IPTG was added to cell culture at 100 *μ*M and the cells incubated for ~16 hours at 30°C until OD_700 _is 0.6, where the cells were then used for tethered cell analysis.

### Tethered cell analysis

*R. sphaeroides *was grown to an OD_700 _of 0.6 and then 4 × 1 ml of cells were harvested. 3 × 1 ml were saved for western blot analysis. The remaining 1 ml was pelted and resuspended in tethering buffer (10 mM Na-HEPES pH 7.2 containing chloramphenicol at 50 *μ*g/ml) and incubated at 30°C for 30 mins.

The cells were then tethered in a humidity chamber by incubation of 10 *μ*l of cell suspension on a coverslip with 1 *μ*l of anti-flagellar antibody for 30 mins.

The coverslip was then loaded onto a flow chamber and tethering buffer passed through for 5 min to remove free cells. After this period the tethered cells were observed under phase contrast at 1000 × magnification. Real time recordings were made on videotape. Tethering buffer with and without Propionate (sodium salt) was passed through the chamber at a rate of 0.09 ml/min.

The video recordings were analysed with the Hobson Bactracker (Hobson Tracking Systems) using the program Arot7. The rotation rate of the cells was measured by detecting the position of the cell every 50 ms. The data obtained were smoothed (100 points), averaged (for as many cells as available - at least 20 per graph) and plotted.

### Western blotting

In order to determine protein concentrations semi quantitative western blots were employed as described previously [26].

### Modelling the chemotaxis pathway in R. sphaeroides

Our model is split into three modules: sensing, transduction and actuation.

#### Sensing

We assumed the same underlying mechanisms for transmembrane (MCP) and the cytoplasmic (Tlp) receptors. The parameters of the Tlp cluster are in brackets and labelled with a tilde superscript. In the following, we list the assumptions for our model, which we adopted from the *E. coli *literature [1,6,27]:

(i) Receptors can be in different states of methylation. For simplicity, we assume that receptors are either methylated or not.

(ii) Only methylated receptors, *R*^*m*^(), can be in an active state, *R*^*a*^().

(iii) Auto-phosphorylation of CheA_2_-P (phosphorylation of CheA_3 _by CheA_4_-P) occurs only when the MCP (Tlp) receptor is active.

(iv) Binding of the ligand to a receptor inhibits its activity.

(v) CheB_1_-P (CheB_2_-P) binds only to active receptors in order to demethylate them.

(vi) CheR_2 _(CheR_3_) binds only to inactive receptors, *R*^*i*^().

(vii) The number of CheR_2 _(CheR_3_) is constant.

We incorporated assumptions (iv), (v) and (vii) into our model as follows:

• We let the number of active receptors depend reciprocally on ligand concentrations *L *() - see below for details.

• We represented the action of CheB_1_-P (CheB_2_-P) through the following term: *K*_2_[*R*^*a*^][*B*_1*p*_].

• We represented the action of CheR_2 _(CheR_3_) by the constant reaction rate *K*_3_().

Using these assumptions, we represented the sensing dynamics by:(1)

where *R*^*t*^() is the total number of receptors, *R*^*i *^= *R*^*t *^- *R*^*a *^(), the *K*_*i *_s are unknown and(6)

where *ε *is a small constant representing disturbances at the input of the cytoplasmic sensing cluster, with nominal amplitude 0.001. We let *α *= 0.1 and *R*^*t *^=  = *μ*M.

We assumed that the cytoplasmic sensing cluster senses extracellular ligand concentrations indirectly; for example,  could be internalised attractants, a byproduct of the internalisation process or a metabolic response to it. For simplicity, we assumed an affine relationship between *L *and . Note that for the red model  is a function of CheY_3_-P and CheY_4_-P concentration levels, reflecting their effect on the activity of the cytoplasmic sensing cluster.

#### Transduction

Table 3 shows the copy numbers of the major proteins involved in the chemotaxis pathway of *R. sphaeroides *[28,29]. The CheA_3_A_4 _copy number is estimated to be the same as the CheA_3 _copy number; this in turn was inferred from neighbouring gene expression because of the lack of a CheA_3_A_4 _antibody. Assuming a cell volume of 0.5 fl for *R. sphaeroides *[30], we obtained the total concentrations of the proteins in *μ*M; this sets the maximum that can be phosphorylated.(7)

**Table 3:** Average copy number of chemotaxis proteins

Number of copies of	value
CheA_2_	26000

CheY_3_	1000

CheY_4_	4000

CheA_3_A_4_	12000

CheY_6_	51500

CheB_1_	23000

CheB_2_	3000

These were determined in microaerobically grown *R. sphaeroides *determined by quantative western blotting [28,29].

where [*A*_2_] denotes the concentration of CheA_2 _and all other expressions in brackets follow this notation. Assuming mass action kinetics and using the dependencies given by (7) and (8), we modeled the transduction part through a set of ODEs:(9)

The model for the transduction part presented in this section is the same for all models except for the magenta model, for which:

where [(*A*_3_*A*_4_)_*i*_] denotes the case when CheA_4_-P cannot phosphorylate CheA_3 _due to the action of CheY_3 _and CheY_4_, and 0.001 ≤ *k*_15 _≤ 1; the latter is to say that the behaviour of the magenta model remains virtually unchanged within this parameter range of *k*_15_.

We obtained the value of *k*_1_, the reaction constant of the auto-phosphorylation of CheA_2_, from *in vitro *experiments in the absence of the influence of receptors. However, when membrane receptors are in their active state they accelerate the auto-phosphorylation of CheA_2_. Thus, we modified the reaction constant to ; that is, we assumed that the *in vitro *reaction rates correspond to the case when receptors are fully active. Similarly, the phosphotransfer from CheA_4_-P to CheA_3 _is accelerated when cytoplasmic receptors are active and we modified the reaction constant to .

The reaction rates were obtained by fitting parameters to data from *in vitro *experiments [16,31] (Table 1).

#### Actuation

We denoted motor activity by *M*_*b *_for the blue, *M*_*r *_for the red, *M*_*g *_for the grey and *M*_*m *_for the magenta model. We assumed some nonspecific interaction, which does not lead to a long lasting binding of proteins to motor sites, either between CheY_6_-P, CheY_3_-P and CheY_4_-P and the motor, or only between CheY_6_-P and the motor. For the blue model we investigated also a different type of CheY motor binding shown below in brackets. This model showed a similar behaviour to the other blue model in simulations and analysis (data not shown) and so we only discussed the findings of the first binding type. Motor activity decreases at a constant rate in the absence of the CheY's, which we assumed to be . We modeled the behaviour of the different models as follows:(16)

The output of the models is flagella activity that we can also observe in tethered cell assays. We used the following heuristic description to convert motor activity into *R. sphaeroides *body rotations, observed in the tethered cell assays (given in rot/sec):(20)

We set *S *= 0.125, which means that saturation occurs at -8 rot/sec.

### Parameter fitting

In order to fit model parameters such that the model represents well the experimental data, we minimised the 2-norm of the vector, whose entries consist of the errors between data and predictions from the discretised version of the ODE models representing the individual chemical reactions investigated *in vitro*. The 2-norm of vector *x *is given by , where *n *is the length of *x*.

In order to fit any remaining model parameters to data from *in vivo *experiments, we simulated the model and minimised the 1-norm of the vector, whose entries are the errors between data and model predictions [32]. The 1-norm of vector *x *is given by |*x*_1_| + |*x*_2_| + ⋯ + |*x*_*n*_|

#### Fitting model parameters for receptor activation

Parameters *K*_1 _- *K*_3 _are unknown and cannot be easily measured by experiments. To determine these we fitted the models to wild type tethering data. We performed tethered cell experiments where we applied 100 *μ*M of attractant for 5 minutes and then removed it. We then ran a simulation of each model (Figure 3). To obtain *K*_*i*_s and s, we minimised the error between computer simulations and data following the online fitting procedure. For simplicity and because we are fitting the models to a single model output only, which is contaminated with noise, we let *K*_*i *_= .

#### Least squares minimisation to fit phosphotransfer parameters

We used least squares minimisation to fit all other parameters from phosphotransfer experimental data. In general, we considered a chemical reaction network with mass action kinetics of the following form:(21)

where *f*(·) ∈ ℝ^*m *^is a vector of known monomials. Let the value of the entries of matrix *A*, which correspond to reaction rate constants, be unknown. What we wished to find is the entries in matrix *A*, given experimental data. For that purpose, we considered the following discrete-time system:(22)

which is the Euler discretisation of (21). Here, each *x*(*t*_*q*_) denotes a measurement at time *t*_*q*_. The set of experimental measurements, which we denote by , was used to fit the unknown entries to *A *such as to minimise the error between the data and the model predictions, which are given by (22). We solved the following optimisation problem which minimises the 2-norm of the error between model prediction and observation (least squares minimisation):(23)

where *p *corresponds to the number of measurements.

Using phosphotransfer data [16,31] and the above method under the constraint that the rates are nonnegative, we obtained values for parameters *k*_1 _- *k*_14 _(Table 1).

#### Online fitting

Because the least squares optimisation is applied to a discretised version of a continuous time model, we applied the *method of steepest descent *online - that is, we minimise the error between simulations of the continuous-time model and the data - to improve the fit using the values obtained before.

### Experiment design

#### Input design

In ordered to discriminate between models by determining an input, which results in the largest difference in the outputs of the models we wished to determine the optimal input frequency. For example consider the red and the blue model, shown in Figure 2, representing different connectivities. The models are given by ordinary differential equations of the form:(24)

Here, *x*_*i *_is the vector with the different states (for example, concentrations of different proteins) of the model *i, i = *1, 2, *f*_*i *_and g_*i *_are functions that represent the different models, and *u *is the input, which can be externally controlled; for example, ligand concentrations.

If *g*_*i*_(*x*_*i*_, 0) = 0 then the two models should have the same equilibrium point x* in order to represent the data equally well:

Moreover, we required that x* is asymptotically stable. The measured output is given by(25)

where *h*_*i *_is the output function. In order to be able to discriminate well between two different competing models the outputs of the two should be as different as possible. Thus, we determined the input that maximised the following difference assuming all other model parameters constant:(26)

Here, *τ *denotes the duration of the experiment. To obtain an input that maximises (27) for a nonlinear system of the form (24)-(25) is difficult. However, if we relax the problem to obtaining the sinusoidal input to the system given by the linearisation of (24)-(25) that maximises (26) then it can be solved systematically as we show in the following.

Consider a linear system(27)

where *A*, *B *and *C *are matrices, whose entries depend on the model parameters, and *u*(*t*) = *a *sin(*ωt*) is a sinusoidal input with angular frequency *ω *and fixed amplitude *a*. System (27) is the so called state space representation of the model in the time domain. It is common in control systems engineering to investigate the behaviour of such a system in dependency of *ω*, which requires to transform the system to the so called frequency domain. If matrix *A *is Hurwitz (stable) then after some transient behaviour output *y *is given by a sinusoidal wave that is, first, phase shifted with respect to *u *and second, has amplitude . Here we are only interested in the maximum of  with respect to *ω*.

We denoted the Laplace transform of *u *and *y *by *U*(*s*) and *Y*(*s*), where *s *is a complex independent variable. Then,

G(*s*) is called the transfer function [33] and is given by

Note that in our analysis the only independent variable is *ω*. We replaced *s *by *jω *(s = *jω*), where . The Bode magnitude plot shows the value of . Thus, the maximum in the plot provides the frequency that will maximise the output to input ratio.

We linearised (9) to (20) to obtain a system of the form given by (27). We determined the frequency of the sinusoidal input that will result in the largest difference between the model outputs as described above. Finally, we simulated the experimental output of this optimal frequency.

#### Initial conditions design

Choosing the change or possible combination of changes that provides best discrimination between the different models is a combinatorial problem and difficult to solve efficiently. However, because for our chemotaxis models the range of possible changes was relatively small, due to being limited by what can be implemented experimentally, we performed a brute force search, checking all possibilities. The five-fold over expression of CheY_4 _under microaerobic growth conditions yielded the best result *in silico *in discriminating between the red and the blue model.

### Robustness of invalidation

In order to assess whether our invalidation conclusions are robust to parameter changes, we performed a sensitivity analysis on our models. We ran 500 simulations allowing parameters *k*_1 _and *k*_14 _to vary by ± 15% (Figure 4C). Even with these variations, we were able reach the same conclusions.

## Authors' contributions

MR provided the information to construct the models and performed all the microbiological experiments and data analysis. EA created and interrogated the models. EA and AH further refined the models. AP and JPA conceived of the study, and participated in its design and coordination, and helped to draft the manuscript. PKM and PEM contributed ideas to the mathematical analysis and aided the project management. MR and EA wrote the manuscript. All authors read and approved the final paper.

## Supplementary Material

Additional file 1 SBML: file containing the models used in this study. Click here for file
